# The impact of assistive technology on burden and psychological well‐being in informal caregivers of people with dementia (ATTILA Study)

**DOI:** 10.1002/trc2.12064

**Published:** 2020-10-07

**Authors:** Anna Davies, Stefano Brini, Shashivadan Hirani, Rebecca Gathercole, Kirsty Forsyth, Catherine Henderson, Rosie Bradley, Lucy Davies, Barbara Dunk, Emma Harper, Natalie Lam, Lynn Pank, Iracema Leroi, John Woolham, Chris Fox, John O'Brien, Andrew Bateman, Fiona Poland, Pete Bentham, Alistar Burns, Richard Gray, Martin Knapp, Emma Talbot, Emma Hooper, Rachel Winson, Bethany Scutt, Victoria Ordonez, Samantha Nunn, Grace Lavelle, Robert Howard, Stanton Newman

**Affiliations:** ^1^ School of Health Sciences City University of London London UK; ^2^ Department of Old Age Psychiatry Institute of Psychiatry London UK; ^3^ School of Health Sciences Queen Margaret University Edinburgh UK; ^4^ South London and Maudsley NHS Foundation Trust London UK; ^5^ Clinical Trial Service Unit Oxford UK; ^6^ Medical Research Council Population Health Research Unit Oxford University Oxford UK; ^7^ Global Brain Health Institute Trinity College Dublin Dublin 2 Ireland; ^8^ Social Care Workforce Research Unit King's College London London UK; ^9^ School of Medicine Health Policy and Practice University of East Anglia Norwich Norfolk UK; ^10^ Department of Psychiatry University of Cambridge Cambridge UK; ^11^ Oliver Zangwill Centre for Neuropsychological Rehabilitation Princess of Wales Hospital Ely UK; ^12^ School of Allied Health Professionals University of East Anglia Norwich Norfolk UK; ^13^ The Barberry Centre Birmingham UK; ^14^ Norfolk and Suffolk NHS Foundation Trust Suffolk UK; ^15^ Lancashire Care NHS Foundation Trust Preston UK; ^16^ Cambridgeshire Community Services NHS Trust Oliver Zangwill Centre Ely UK; ^17^ Division of Psychiatry University College London London UK; ^18^ Policy and Evaluation Centre London School of Economics and Political Science London UK; ^19^ Oxford Health NHS Foundation Trust Warneford Hospital Headington Oxford UK; ^20^ Centre for Clinical Brain Sciences University of Edinburgh Edinburgh UK

**Keywords:** assistive technology, caregiver burden, dementia, informal caregiver, mental health, telecare

## Abstract

**Introduction:**

Assistive technology and telecare (ATT) may alleviate psychological burden in informal caregivers of people with dementia. This study assessed the impact of ATT on informal caregivers’ burden and psychological well‐being.

**Methods:**

Individuals with dementia and their informal caregivers were recruited to a randomized‐controlled trial assessing effectiveness of ATT. Caregivers were allocated to two groups according to their cared‐for person's randomization to a full or basic package of ATT and were assessed on caregiver burden, state anxiety, and depression. Caregivers’ data from three assessments over 6 months of the trial were analyzed.

**Results:**

No significant between‐ or within‐group differences at any time point on caregivers’ burden, anxiety, and depression levels were found.

**Discussion:**

Full ATT for people with dementia did not impact caregivers’ psychological outcomes compared to basic ATT. The length of follow up was restricted to 6 months.

## 1 INTRODUCTION

Caring for a person with dementia is associated with poor psychological and physical well‐being^1^ placing greater psychological burden on the caregiver than caring for individuals with other chronic conditions.^2^ Interventions to prevent poor psychological outcomes and institutionalization of the person with dementia have been developed. Psychological support interventions that target informal carers directly can be delivered face‐to‐face or over the telephone.^3^ A systematic review of 40 studies found that interventions including a social component, with or without a cognitive component, were more effective in improving psychological well‐being than interventions without such components.^4^ Small sample sizes and differences in the types of interventions might explain differences in study outcomes. More than 200 interventions for caregivers have been tested in randomized trials and found to have some efficacy on caregivers’ outcomes.^5^ Telephone‐based interventions to support caregivers communicate between patient and the health‐care systems appear to be effective in improving outcomes.^6^ A recent meta‐analysis has identified that telecare can improve health outcomes in caregivers.^7^

An alternative to interventions targeting the caregivers directly are those aiming to remotely monitor and manage the care recipient. Information communication technologies, such as those collecting, capturing, storing, processing, transmitting, exchanging, and presenting information, and/or communication, appear to facilitate delivery and access of health care to individuals with a chronic disease.^8^, ^9^ Assistive technology and telecare (ATT) involves installing equipment to manage the risks of living at home. Some ATT devices continuously, automatically, and remotely monitor for real‐time emergencies and lifestyle changes;^10^, ^11^ others “stand alone” (eg, electronic reminders, key safes). While directed at the care recipient, these may also impact caregiver outcomes by improving sleep and reducing worry and stress by providing alerts to serious incidents such as falls, cooking accidents, or wandering, thus enabling appropriate and timely intervention. A systematic review of seven studies, three of which were of caregivers for individuals with dementia, showed that telecare exerts a positive effect on caregiver stress and strain.^12^ The reports that included caregivers of dementia care recipients were not peer‐reviewed publications, and as such, caution in interpreting findings from this systematic review is warranted. Overall, however, findings do suggest a trend favoring the application of ATT for caregivers and care recipients, which needs to be investigated further.

We conducted a pragmatic randomized controlled trial (RCT) as part of the larger Assistive Technologies and Telecare to Maintain Independent Living At Home (ATTILA) trial.^13^ The ATTILA trial examined the clinical and cost‐effectiveness of ATT in supporting people with dementia to continue living safely within their own homes and the impact of the intervention on caregiver psychological outcomes.^13^ This article reports on the impact of the intervention on informal caregiver outcomes.^13^ The aim of this substudy of the ATTILA trial was to compare the effect of a full ATT versus basic ATT package for people with dementia on their caregivers’ psychological outcomes.

## 2 METHODS

### 2.1 Design

This was a substudy of the ATTILA RCT and used a quasi‐experimental design, examining the effect of receipt of ATT services on psychological outcomes of carers of people with dementia^13^ (Trial Protocol Reference ISRCTN86537017). Participants in the current study were informal caregivers of people with cognitive difficulties or dementia who had been recruited to the ATTILA trial.^13^

### 2.2 Participants

In the ATTILA trial, participants were people with a diagnosis of dementia or cognitive difficulties sufficient to suggest dementia, who met English Social Services’ eligibility criteria for Fair Access to Care Services (an eligibility framework in England for prioritizing the use of adult social care resources), were living in the community, and had a working telephone line. Exclusion criteria were current receipt of an ATT intervention (except for the provision of non‐monitored smoke and carbon monoxide alarms, key safes, and pendant alarms) or previous installation of ATT that had not been used, unlikely to comply with long‐term follow‐up, participation in another interventional dementia trial, or had an identified urgent need for a home care package due to immediate and severe risk to participant or others. Informal caregiver participants were adults, who could be co‐resident or non‐resident with the trial participant. The caregiver remained in the trial for the full 104‐week trial duration or until their care recipient left due to death or institutionalization or withdrawal from the trial.

### 2.3 Intervention and control conditions

Informal caregivers were allocated to the intervention or control arm according to the randomization group of their cared‐for person. Participants with dementia in the ATTILA trial were randomized to one of two conditions: (1) Intervention: a semi‐structured needs assessment for ATT by a health or social care professional, followed by installation of ATT devices and response services as indicated by the assessment, or (2) Control: a semi‐structured needs assessment for ATT by a health or social care professional, followed by installation of devices restricted to a non‐monitored smoke or carbon monoxide alarm, key safe, and pendant alarm where indicated.

### 2.4 Sample size

The sample size was estimated on the expected effect size of the intervention on the primary outcome (ie, time to institutionalization) for the ATT recipients. No required number of participants was identified for the caregiver sample.

### 2.5 Procedure

Outcome rating scales were completed by caregivers at the same time points as scheduled data collection for their care recipient: baseline (0 weeks), 12, 24, 52, and 104 weeks. Data were collected on the care recipients and their respective caregivers. Caregivers completed the baseline data collection at home, with or without the assistance of the data collection assistants. Further assessments were mailed to caregivers or completed at the care recipients’ follow‐up appointments.

**HIGHLIGHTS**

- Informal caregivers of people with dementia have been found to have poor psychological well‐being.
- We investigated the impact of a full package assistive technology and telecare (ATT) implemented for the cared‐for person on informal caregivers’ psychological well‐being.
- The psychological well‐being of informal caregivers of people with dementia receiving a full package ATT did not differ from that of caregivers of people with dementia not in receipt of a full package of ATT.

**RESEARCH IN CONTEXT**

1. Systematic review: Electronic databases were searched for systematic reviews of interventions for informal caregivers of people with dementia. Several reviews assessed interventions to improve carer psychological outcomes but did not investigate second and third generation assistive technology and telecare (ATT). Our published systematic review identified three studies implementing telecare for a person with dementia and assessing informal caregivers’ outcomes, of which none were peer‐reviewed or randomized controlled trials (RCTs).
2. Interpretation: To our knowledge, ATTILA (Assistive Technologies and Telecare to Maintain Independent Living at Home Yrial) is the first RCT to assess the effectiveness of ATT for a person with dementia on informal carers’ psychological well‐being. We have assessed its impact in a large sample and provide insight into the short‐term impact of its installation on psychological well‐being among caregivers.
  3. Future directions: To confirm our findings, future studies should identify the minimum sample size needed to detect an effect of ATT on informal carer outcomes and should carry out longer follow‐up assessments to determine whether carer benefits are manifest later.

### 2.6 Descriptive data

Data about the caregiver, their caring responsibilities, and their relationship to the participant were collected, including: (1) caregiver age, (2) frequency of caring responsibility (lives with the care recipient, visits once per day, or visits less than once per day), (3) who lived with the care recipient (spouse or partner, care recipient lives alone, or other). Data about the severity of the care recipient's dementia symptoms were captured using the Standardized Mini‐Mental State Examination (SMMSE).^14^

### 2.7 Caregiver outcome data

Data were collected about caregiver outcomes on three scales at each time point:

1. Caregiver burden: The Zarit Burden Interview^15^ is a 22‐item scale assessing burden of caregiving. Participants respond on a 5‐point Likert‐type scale ranging from 0 (never) to 4 (always), to generate a single score with higher scores indicating greater burden. Scores 0 to 20 indicate little or no burden, 21 to 40 mild to moderate burden, 41 to 60 moderate to severe burden, and 61 to 88 indicating severe burden.
2. Depression: Centre for Economic Studies Depression Scale‐10 (CES‐D‐10): A 10‐item scale. Participants respond on a 4‐point Likert‐type scale ranging from 0 (rarely/none of the time) to (3) all of the time. A single score, ranging from 0 to 30, is calculated. A score ≥10 indicates depression.
3. State Anxiety: Short form of the state scale of the Spielberger State‐Trait Anxiety Inventory (STAI):^16^ A 6‐item list on which participants rate anxiety symptoms on a four‐point Likert‐type scale ranging from 1 (not at all) to 4 (very much). A single score is calculated ranging from 20 to 80 points; higher scores represent greater anxiety. A “normal” score is 34 to 36 points.

### 2.8 Data analysis

We analyzed the data with the Statistical Package for the Social Sciences version 25 (alpha level = .05). Normality of the data was examined by visual inspection of the histograms and conducting the Shapiro‐Wilk test of normality. To establish the structure of the Zarit Burden Interview in this sample a principal component analysis (PCA) with an Oblimin rotation was performed. We used the Kaiser‐Meyer‐Olkin test to check the suitability of the data for PCA, followed by inspection of a scree plot to determine the number of factors.

### 2.9 Selection of cases/timepoints for inclusion in analyses

There were several sources of attrition across time points including loss to follow‐up, death, or institutionalization of the care recipient. Because rates of attrition at the later time points reached approximately 50% by week 104, analysis of the caregiver sample was restricted to baseline, week 12, and week 24. Intention to treat analyses were conducted.

### 2.10 Imputation

To account for missing data across demographic variables and outcomes, we conducted multiple imputations for baseline only, by including all predictors to fill the missing data. We used data from all three examined time points (baseline, week 12, and week 24) within the same multiple imputation model. We produced 10 imputed datasets (*m* = 10); each of the multiply imputed datasets was analyzed as usual, after which the 10 sets of results produced for each analysis were combined using Rubin's rules.^17^, ^18^, ^19^

### 2.11 Descriptive data, randomization, and loss to follow‐up analyses

Means and standard deviations were calculated for continuous data and frequencies and percentages for categorical data. We conducted linear mixed modeling (LMM) to analyze between‐group differences, change over time, as well as interaction effects of group and time. An initial set of analyses was conducted to examine the assumption that within‐participant scores are highly correlated by calculating the intraclass correlation. The second set of models included covariates. Time was entered as a fixed effect for each LMM with participants’ identification number as random effect with the default variance components structure.

In addition to the main effects of group and time, the effects of the time–group interaction were examined and interpreted where a significant interaction term indicating differential treatment effectiveness was found. The decomposition of interaction effects for (1) group differences within each time point and (2) changes over time within each group individually was examined. Significant effects were investigated using pairwise comparison with the estimated marginal means. The 95% confidence intervals around the estimated marginal means on each outcome for each group were also calculated. All LMM analyses in each section were adjusted for each of the demographic variables presented in Table 1. Alpha level was set at 0.05.

**TABLE 1:** Caregiver and care recipient demographics (*N* = 495)

Sample statistics					
*Variable*	Mean	SEM	95% CI		*P*
Age					
Total sample	62.5	0.6	61.3	63.7	
Control	62.1	0.85	60.4	63.7	.455
Intervention	63.0	0.85	61.3	64.6	
**Care recipient SMMSE** ^a^					
Total sample	17.8ss	0.3	17.2	18.4	
Control	17.0	0.43	16.2	17.8	.006
Intervention	18.6	0.41	17.8	19.4	
Frequencies					
*Variable*	Frequency	%	Valid %		
**Living status**					
Living alone	229	46.3	46.3		
Living with spouse/partner	195	39.4	39.4		
Other	71	14.3	14.3		
Total	495	100	100		
**Caregiver visits**					
Caregiver visits at least once per day	121	24.4	24.4		
Caregiver visits less than once per day	134	27.1	27.1		
Live‐in caregiver	240	48.5	48.5		
Total	495	100	100		

Abbreviations: CI, confidence intervals; SEM, standard error of the mean; SMMSE, Standardized Mini‐Mental State Examination.

^a^ SMMSE, Standardized Mini‐Mental State Examination scores of the care recipients.

## 3 RESULTS

### 3.1 Participants

Four hundred ninety‐five people with dementia and, where available, their caregivers, were recruited to the trial. Of participating caregivers, 354 provided data on age (control *n *= 182, intervention *n *= 172) and on SMMSE scores for the person with dementia. The remaining 141 missing data for age and SMMSE scores were imputed. Baseline caregiver and care recipient demographic characteristics are summarized in Table 1 and baseline scores for each outcome are summarized in Table 2.

**TABLE 2:** Participants’ baseline scores for each outcome (whole sample: *N* = 495)

ZBI: Total score		Mean	SE	95% CI	
Baseline	Control	29.6	1.36	26.9	32.3
	Intervention	29.3	1.44	26.4	32.1
**ZBI Component 1: Negative Appraisal of Caring**					
Baseline	Control	13.8	0.67	12.5	15.1
	Intervention	14.0	0.70	12.6	15.4
**ZBI Component 2: Adequacy as a Caregiver**					
Baseline	Control	3.8	0.25	3.3	4.3
	Intervention	3.9	0.26	3.4	4.4
**ZBI Component 3: Caregiver Burden and Strain**					
Baseline	Control	7.8	0.50	6.8	8.8
	Intervention	7.4	0.53	6.4	8.5
**CES‐D‐10—Depression**					
Baseline	Control	9.6	0.56	8.5	10.7
	Intervention	8.7	0.59	7.5	9.8
**STAI‐6—State Anxiety**					
Baseline	Control	40.3	1.22	37.9	42.7
	Intervention	39.7	1.28	37.2	42.2

Abbreviations: CES‐D‐10, Center for Epidemiological Studies Depression Scale Revised; CI, confidence intervals; SE, standard error; STAI‐6, Spielberger State‐Trait Anxiety Inventory; ZBI, Zarit Burden Interview.

### 3.2 Caregiver burden

The Zarit Burden Inventory (ZBI) was analyzed as total score, and as three‐component factors following a PCA. The three components were defined as: (1) Component 1: Negative appraisal of the care partner role, (2) Component 2: Adequacy as a care partner, (3) Component 3: Caregiver burden and strain. Total scores and the three‐component scores for the ZBI were not significantly different between the control and intervention group at 12 or 24 weeks. There were no significant within‐group or interaction effects across all time points (see Table 2).

We also conducted post hoc subgroup analyses among live‐in caregivers, and in caregivers who were the spouse or partner of the cared‐for person, in whom we might expect poorer psychological well‐being and levels of burden. Neither of these subgroup analyses revealed differences between the two groups in any of these outcomes.

### 3.3 Caregiver depression and anxiety

Scores for CES‐D‐10 (depressed mood) were not significantly different between the control and intervention group and there were no significant interaction effects across all time points. Similarly, scores for the STAI‐6 (anxiety) did not significantly differ between the control and intervention group and no significant interaction effects were found. Parameter estimates and adjusted mean scores for each group at each time point are presented in Table 3.

**TABLE 3:** ZBI: Burden for all caregivers for total score and for three principal components, CES‐D‐10, and STAI‐6

ZBI: Total score		**F‐Value**	**df1**	**df2**	***P***	
	Time	0.472	2	1438503	.623	
	Group	0.036	1	161355	.849	
	Interaction	0.172	2	2228089	.842	

		**Mean**	**SE**	**95% CI**		**MD (95% CI)**
Baseline	Control	29.6	1.36	26.9	32.3	0.33 (–3.56, 4.22)
	Intervention	29.3	1.44	26.4	32.1	
Week 12	Control	29.7	1.41	27.0	32.5	0.27 (–3.74, 4.28)
	Intervention	30.0	1.48	27.1	32.9	
Week 24	Control	30.0	1.43	27.2	32.7	0.30 (–3.74, 4.34)
	Intervention	29.7	1.48	26.7	32.6	
ZBI Component 1: Negative Appraisal of Caring						

		**F‐Value**	**df1**	**df2**	***P***	
	Time	0.127	2	645845	.881	
	Group	0.042	1	654751	.838	
	Interaction	0.2	2	4649804	.819	

		**Mean**	**SE**	**95% CI**		**MD (95% CI)**
Baseline	Control	13.8	0.67	12.5	15.1	0.2 (–1.7, 2.1)
	Intervention	14.0	0.70	12.6	15.4	
Week 12	Control	14.3	0.70	13.0	15.7	0.1 (–1.8, 2.1)
	Intervention	14.2	0.73	12.8	15.6	
Week 24	Control	14.3	0.70	13.0	15.7	0.2 (–1.8, 2.1)
	Intervention	14.2	0.73	12.8	15.6	
ZBI Component 2: Adequacy as a Caregiver						

		**F‐Value**	**df1**	**df2**	***P***	
	Time	1.259	2	318819	.284	
	Group	0.144	1	37476	.704	
	Interaction	0.653	2	50769	.52	

		**Mean**	**SE**	**95% CI**		**MD (95% CI)**
Baseline	Control	3.8	0.25	3.3	4.3	0.1 (–0.6, 0.8)
	Intervention	3.9	0.26	3.4	4.4	
Week 12	Control	3.9	0.27	3.3	4.4	0.2 (–0.6, 0.9)
	Intervention	4.1	0.27	3.5	4.6	
Week 24	Control	3.9	0.27	3.3	4.4	0.1 (–0.6, 0.9)
	Intervention	3.7	0.28	3.2	4.3	
ZBI Component 3: Caregiver Burden and Strain						

		**F‐Value**	**df1**	**df2**	***P***	
	Time	1.696	2	250490	.183	
	Group	0.03	1	272088	.863	
	Interaction	1.657	2	578798	.191	

		**Mean**	**SE**	**95% CI**		**MD (95% CI)**
Baseline	Control	7.8	0.50	6.8	8.8	0.3 (–1.1, 1.8)
	Intervention	7.4	0.53	6.4	8.5	
Week 12	Control	7.5	0.53	6.5	8.6	0.5 (–1.0, 2.0)
	Intervention	8.0	0.54	6.9	9.1	
Week 24	Control	7.7	0.53	6.6	8.7	0.1 (–1.4, 1.6)
	Intervention	7.8	0.55	6.7	8.8	
CES‐D‐10—Depression						

		**F‐Value**	**df1**	**df2**	***P***	
	Time	1.726	2	935042	.178	
	Group	0.282	1	341074	.596	
	Interaction	0.595	2	830859	.551	

		**Mean**	**SE**	**95% CI**		**MD (95% CI)**
Baseline	Control	9.6	0.56	8.5	10.7	0.9 (–0.7, 2.5)
	Intervention	8.7	0.59	7.5	9.8	
Week 12	Control	9.8	0.59	8.6	10.9	0.7 (–1.0, 2.3)
	Intervention	9.1	0.61	7.9	10.3	
Week 24	Control	9.7	0.59	8.5	10.8	0.4 (–1.3, 2.0)
	Intervention	9.3	0.61	8.1	10.5	
STAI—State Anxiety						

		**F‐Value**	**df1**	**df2**	***P***	
	Time	1.11	2	4613788	.329	
	Group	0.539	1	2187757	.463	
	Interaction	0.713	2	896778.4	.49	

		**Mean**	**SE**	**95% CI**		**MD (95% CI)**
Baseline	Control	40.3	1.22	37.9	42.7	0.6 (–2.9, 4.0)
	Intervention	39.7	1.28	37.2	42.2	
Week 12	Control	39.9	1.30	37.4	42.5	0.3 (–3.4, 4.0)
	Intervention	40.2	1.34	37.6	42.8	
Week 24	Control	40.1	1.33	37.5	42.7	1.1 (–2.6, 4.8)
	Intervention	41.2	1.36	38.5	43.9	

Abbreviations: CES‐D‐10, Center for Epidemiological Studies Depression Scale Revised; CI, confidence intervals; *df*, degrees of freedom; MD, mean difference; SE, standard error; STAI‐6, Spielberger State‐Trait Anxiety Inventory; ZBI, Zarit Burden Interview.

We also conducted post hoc subgroup analyses among live‐in caregivers, and in caregivers who were the spouse or partner of the cared‐for person, in whom we might expect poorer psychological well‐being and levels of burden. Neither of these sub‐group analyses revealed differences between the two groups in any of these outcomes.

## 4 DISCUSSION

The impact of caring for someone with dementia on informal caregivers’ health and well‐being has led to the development of interventions to reduce caregivers’ burden.^20^ These interventions may have a broader impact because alleviating caregivers’ burden and psychological difficulties may reduce the likelihood of the care recipient being institutionalized, resulting in lower social and health‐care costs. In this substudy of the ATTILA trial, we compared the impact of deploying the full or basic ATT package in the home of the people with dementia on the psychological outcomes of their caregivers (caregiver burden, depression, and anxiety) in the first 24 weeks following its installation.

Mean scores of caregiver burden, depression, and state anxiety did not differ between the caregivers of trial participants in the intervention and control groups at follow‐up. Subanalyses on live‐in caregivers and those who were the spouse or partner of the cared‐for person also revealed no effects of the intervention on caregiver burden or psychological well‐being. It is notable that the caregiver burden levels, depression, and anxiety remained stable during the course of the study. Although this study was not conducted as a non‐inferiority trial, the data suggest no negative impact of receiving the ATT intervention on caregiver burden and psychological outcomes.

One explanation for the lack of impact on these outcomes is the relatively low baseline levels of burden, depression, and state anxiety.^21^ Mean burden in the intervention and control group for the overall sample and the examined subgroups were in the mild to moderate range. Similarly, mean levels of depression in this sample were below the clinically relevant threshold on the CES‐D‐10 scale, for which a score >10 indicates depression. For state anxiety, mean scores on this scale at baseline were 40.3 (standard error 1.22) and 39.7 (standard error 1.28) for the control and intervention group, respectively. Therefore, participants might have had sufficiently high levels of anxiety at baseline to benefit from the intervention. Previous studies have indicated higher levels of depression and anxiety at baseline in their study populations (see, eg, Blom et al.^22^), and a recent study using the same instrument for assessing depression found higher scores, above the clinically relevant threshold, in their sample.^23^

Alternatively, it is possible that the effects of the intervention may have been limited in effecting change in these outcomes. Interventions specifically targeting caregivers may be more effective than those aiming to support the cared‐for person. Meta‐analyses indicate that caregiver‐directed interventions have demonstrated effectiveness on average in reducing depression; effective interventions include Cognitive Behavioral Therapy, cognitive reframing, and educational interventions.^20^, ^24^, ^25^, ^26^ Therefore, to optimize the benefits of the installation of ATT for both the care recipient and the caregiver, it may be important to provide additional caregiver‐directed practical and psychosocial support. Effective and potentially low‐burden and low‐cost modes of delivery of these interventions include the use of telephone and internet.^27^, ^28^

In the current sample, mean SMMSE scores indicated moderate levels of cognitive impairment in the cared‐for participant sample. There is some evidence to indicate that the severity of dementia is related to levels of depression and anxiety, with only severe dementia leading to caregivers having high levels of depression and anxiety,^29^, ^30^ although this relationship has not always been confirmed.^31^ Furthermore, while we observed baseline between‐group differences in SMMSE scores, the magnitude of this difference was marginal with fewer than two points between the control and intervention group. Additionally, in our analyses, we adjusted for SMMSE scores at baseline. It is also possible that disease severity in the care recipient was not sufficiently severe to produce high burden, depression, or anxiety scores at baseline in the caregivers such that they may have been reduced by the intervention. It is of note that a small but significant difference was found between the two groups with those receiving ATT having higher scores on the MMSE.

While the care recipients had been diagnosed with dementia, they were of mixed etiology and severity. Furthermore, there was a low risk of wandering in the sample at baseline, with 72% of participants with dementia being classified as being at low risk of wandering, and half of participants identified as having a low safety risk in their own home. It is possible that the effects of ATT on caregivers’ burden might be related to varying levels of cognitive impairment in the care recipient^32^ and the type of dementia.^33^ Moreover, different dementia types manifest varying levels of behavioral problems. Thus caring for someone with frontotemporal dementia, which tends to present with greater behavioral problems than Alzheimer's disease (AD), for example, may impact caregiver's burden and depression differently.^33^, ^34^ Identifying what type of dementia etiology (AD, vascular dementia, etc.) may inform the selection of the type of intervention that should be applied to alleviate the caregiver's burden.^32^

A further potential explanation of the lack of impact of the intervention may be the limited fidelity of technology deployment in relation to the recommendations arising from the needs assessment.^35^ A moderate correlation was found between the intervention ATT deployed and the needs of the person with dementia. If the ATT did not address the problems experienced by the individual and their caregiver, it can be expected to have had limited impact on the carers’ outcomes.

### 4.1 Strengths, limitations, and suggestions for future research

This study provides the first insight into the potential impact of ATT interventions for people with dementia on outcomes for their informal caregivers. Because of the design of the trial, after care‐recipients had left the study due to death or institutionalization, their informal caregivers were no longer followed up. Thus, the attrition rate in caregivers after 24 weeks was considerable, precluding analysis of caregiver data after this time point. It is possible that any effects of ATT on caregivers’ psychological well‐being may take some time to manifest, beyond the limited time scale in this study. Furthermore, the sample size for the ATTILA study was based on the study primary outcome (time to institutionalization) rather than on caregivers’ outcomes. It is possible that our analyses were statistically underpowered to detect intervention effects.

Caregivers in this study had only limited characterization such that age, sex, and cognitive ability were not assessed. While it is reasonable to assume that randomization would have ensured appropriate distribution of these characteristics, such that they would be evenly distributed across the two groups, it was not possible to examine these characteristics statistically.

In light of the limitations above, future work should determine the minimum sample size to detect an effect of the ATT intervention based on expected effect size for caregiver outcomes. It may well be that longer follow‐up times and additional support interventions for caregivers are necessary to effect benefits for caregivers’ outcomes. It may also be fruitful to examine at which stage of the condition assistive technologies should be introduced so that the person with dementia and caregiver can derive the maximum benefit; and to examine which ATT devices are most useful at different stages of dementia.

### 4.2 Conclusions and implications for practice

This study provides insight into the potential impact on caregiver burden and psychological well‐being of providing people with dementia with a comprehensive package of ATT compared to a basic package. No impact of ATT on caregiver burden, depression, and anxiety was identified. Thus, interventions aiming to specifically target caregiver well‐being alongside the deployment of ATT may be important for delaying institutionalization and associated costs. Effective interventions to reduce the impact of caregiving may include caregiver‐directed psychological techniques as well as ensuring that caregivers have an appropriate understanding of the role of ATT, and scope for change when using ATT.

#### CONFLICTS OF INTEREST

P.B. has received financial support from TauRx Therapeutics. J.O.B. has received financial support from TauRx, GE Healthcare, Avid/Lilly, and Eisai. No other author has any conflict of interest.
